# Conversion surgery for stage IV gastric cancer: a multicenter retrospective study

**DOI:** 10.1186/s12893-022-01874-8

**Published:** 2022-12-14

**Authors:** Yosuke Kano, Hiroshi Ichikawa, Takaaki Hanyu, Yusuke Muneoka, Takashi Ishikawa, Masaki Aizawa, Atsushi Matsuki, Hiroshi Yabusaki, Takeo Bamba, Satoru Nakagawa, Kazuaki Kobayashi, Shirou Kuwabara, Shigeto Makino, Yasuyuki Kawachi, Tetsuya Naito, Tatsuo Tani, Hiroshi Hirukawa, Tetsuya Tada, Yoshifumi Shimada, Jun Sakata, Toshifumi Wakai

**Affiliations:** 1 Division of Digestive and General Surgery, Niigata University Graduate School of Medical and Dental Sciences, 1-757 Asahimachi-dori, Chuo-ku, Niigata, 951-8510 Japan; 2 Department of Surgery, Shibata Prefectural Hospital, 1-2-8 Hon-cho, Shibata, Niigata 957-8588 Japan; 3 Department of Gastroenterological Surgery, Niigata Cancer Center Hospital, 2-15-3 Kawagishi-cho, Chuo-ku, Niigata, 951-8566 Japan; 4 Department of Surgery, Niigata City General Hospital, 463-7 Shumoku, Chuo-ku, Niigata, 950-1197 Japan; 5 Department of Surgery, Nagaoka Chuo General Hospital, 2041 Kawasaki-cho, Nagaoka, Niigata 940-0861 Japan; 6 Division of Digestive and General Surgery, Japanese Red Cross Nagaoka Hospital, 2-297-1 Senshu, Nagaoka, Niigata 940-2085 Japan; 7 Department of Surgery, Tachikawa General Hospital, 1-24 Asahioka, Nagaoka, Niigata 940-8621 Japan

**Keywords:** Gastric cancer, Stage IV, Conversion surgery

## Abstract

**Background:**

Recent improvements in systemic chemotherapy have provided an opportunity for patients with stage IV gastric cancer (GC) to undergo conversion surgery (CS). The aim of this study was to evaluate the long-term outcomes of patients who underwent CS and to elucidate the prognostic factors for CS in stage IV GC.

**Methods:**

A total of 79 patients who underwent CS with the aim of R0 resection for stage IV GC at six institutions from January 2008 to July 2019 were enrolled. We retrospectively reviewed the clinicopathological data and prognosis.

**Results:**

Of the 79 patients, 23 (31.1%) had initially resectable disease (IR) before chemotherapy, defined as positive for cancer on peritoneal cytology (CY1), resectable hepatic metastasis, or para-aortic lymph node No. 16a2/b1 metastasis. Of the 56 remaining patients with primary unresectable disease, 39 had peritoneal dissemination. R0 resection was accomplished in 63 patients (79.7%). The 3-year OS rates for patients with IR and unresectable disease were 78.3% and 44.5%, respectively. Multivariate analysis showed that IR (*P* = 0.014) and R0 (*P* = 0.014) were statistically significant independent prognostic factors for favorable OS. Among patients with peritoneal dissemination alone, OS was significantly better for patients with R0 resection than for patients with R1/2 resection, with the 3-year OS rates of 65.5% and 23.1%, respectively (*P* = 0.011).

**Conclusions:**

CS is a treatment option for selected patients with stage IV GC. Patients with IR and patients who achieve R0 resection may obtain a survival benefit from CS.

**Supplementary Information:**

The online version contains supplementary material available at 10.1186/s12893-022-01874-8.

## Background

Gastric cancer (GC) is the third most common cause of cancer-related death worldwide [1]. The standard treatment for stage IV GC is systemic chemotherapy, and patients are currently not considered surgical candidates unless required as palliative surgery for stenosis or bleeding. This policy is supported by the results of the REGATTA phase III study, in which gastrectomy followed by chemotherapy did not confer a survival benefit compared with chemotherapy alone in patients with advanced GC with a single non-curable factor [2].

In some patients with stage IV GC who respond well to chemotherapy, conversion surgery (CS), defined as gastrectomy with or without metastasectomy with the goal of R0 resection, may become an option. However, whether continuing chemotherapy or converting to surgery is a better option for such patients is unclear. One factor to consider is that long-term chemotherapy can lead to chemoresistance and be accompanied by severe adverse effects, resulting in tumor progression and worsening quality of life. CS with R0 resection may be a feasible option to prevent such outcomes. Recent studies have demonstrated increased survival of stage IV GC patients who underwent CS with the aim of R0 resection after responding to chemotherapies, molecularly targeted therapies and immunotherapies [3–11]. Some studies have reported that stage IV GC patients who underwent CS had better survival rates than those who continued with chemotherapy alone [3, 5, 12], suggesting that CS is a promising option for improving the survival for stage IV GC patients.

Stage IV GC consists of a mixture of disease with various metastatic behaviors and different biology. Yoshida et al. classified patients with stage IV GC into four categories based on oncosurgical treatment strategies [13]. Although some studies have examined surgical outcomes of stage IV GC after chemotherapy based on the Yoshida classification, the risk factors and outcomes of CS for such patients have not yet been fully examined. The aim of this retrospective multicenter study was to evaluate the long-term outcomes of CS in stage IV GC patients, and to elucidate prognostic factors that can be used to identify patients who might be good candidates for CS.

## Methods

### Study design and ethics

This retrospective observational study included patients seen at Niigata University Medical and Dental Hospital and five affiliated institutions. This study was approved by the Human Ethics Review Committee of Niigata University (#2018-0137). The need for written informed consent was waived due to the retrospective observational nature of the study, and brief information on this study was disclosed on the Nigata University website to ensure patients had an opportunity to refuse their participation in the study (opt-out method).

### Patients

We retrospectively reviewed the medical records of patients who were diagnosed with stage IV GC and who converted to surgery after chemotherapy with the aim of R0 resection between January 2008 and December 2018. Inclusion criteria were: Eastern Cooperative Oncology Group performance status 0–2, histologically confirmed adenocarcinoma, clinical or pathological diagnosis of M1 lesion, no synchronous cancer, and no history of gastrectomy. We excluded patients who converted to surgery due to discontinuation of chemotherapy because of adverse effects.

### Tumor stage and classification for stage IV GC

Tumor stage was evaluated by gastrointestinal endoscopy and computed tomography (CT), and was classified according to the Japanese classification of gastric carcinoma [14]. Positive for cancer on peritoneal cytology (CY1) and peritoneal dissemination were pathologically proven by staging laparoscopy or exploratory laparotomy before starting the initial chemotherapy. Magnetic resonance imaging, positron emission tomography-CT, and radionuclide bone scintigraphy were performed as needed to diagnose distant metastasis.

Stage IV GC patients were classified into four categories according to the classification reported by Yoshida et al. [13]. Category 1 included patients with CY1, para aortic lymph node (PAN) metastasis of No. 16a2 and/or 16b1, and a solitary liver metastasis < 5 cm in diameter. Category 2 included patients who had distant metastasis regarded as technically and oncologically unresectable tumor other than peritoneal dissemination. Category 3 include patients with macroscopic peritoneal dissemination without other distant metastasis. Category 4 include patients with macroscopic peritoneal dissemination with other distant metastasis. In this study, we defined category 1 as initially resectable (IR) and categories 2–4 as unresectable (UR) disease.

### Treatment strategy and histopathological examination

The treatment regimen, including duration and dose of perioperative chemotherapy and timing of surgery, were at the physician’s discretion. We defined CS as surgery with the aim of R0 resection for stage IV GC after chemotherapy. Lymph node dissection was classified according to the Japanese gastric cancer treatment guidelines 2014 (ver.4) [15]. Postoperative complications were evaluated according to the Clavien–Dindo classification [16, 17]. Postoperative follow-up included evaluation of serum carcinoembryonic antigen and CA19-9 levels every 3 months and CT at least every 3–6 months for 5 years after surgery. Regarding histological tumor type: papillary and tubular adenocarcinomas were classified as either differentiated or poorly differentiated types; and signet ring cell and mucinous adenocarcinomas were classified as undifferentiated types. In this study, the histological response to chemotherapy was classified as: grade 0, no evidence of effect; grade 1, viable tumor cells remain in 1/3–2/3 of the tumorous area; grade 2, viable tumor cells remain in less than 1/3 of the tumorous area; and grade 3; no viable tumor cells remain [14].

### Endpoints and statistical analysis

The endpoints of this study were overall survival (OS) and prognostic factors for OS. OS was calculated from the start of initial chemotherapy to the date of death or of the last follow-up visit. OS was calculated by the Kaplan–Meier method and evaluated using the log-rank test as a univariate analysis. In the multivariate analysis, the factors with P < 0.10 in the univariate analyses were selected for the multivariate analysis and variable selection was performed by step-backward method. Hazard ratios (HRs) are presented with 95% confidence intervals (CIs). *P* < 0.05 was considered to be significant. All statistical analyses were performed with SPSS version 24.0 for Windows (IBM Inc., Chicago, IL, USA).

## Results

### Clinicopathological and chemotherapeutic features of the study population

A total of 79 patients were enrolled in this study. Table 1 shows the patient characteristics at diagnosis. According to the metastatic status, 23 patients (29.1%) were classified as category 1 with IR stage IV GC before chemotherapy. Of the remaining 56 patients with UR, 17 (21.5%) were classified as category 2, 33 (41.8%) as category 3, and 6 (7.6%) as category 4 before chemotherapy. Category 2 included distant lymph node metastasis other than No. 16a2 and/or 16b1 in 8 patients, multiple liver metastasis in 4 patients, distant lymph node metastasis other than No. 16a2 and/or 16b1 and multiple liver metastasis in 2 patients, multiple liver metastasis and lung metastasis in 2 patients, and lung metastasis in a patient.

**Table 1:** Patient characteristics at diagnosis

Variable		No. of patients (%)^a^	
Age (years), median (IQR)		63	(54–69)
Sex			
	Men	51	(64.6)
	Women	28	(35.4)
Tumor location			
	Upper third	30	(38.0)
	Middle third	25	(31.7)
	Lower third	13	(16.5)
	Whole of the stomach	11	(13.9)
Histological type			
	Differentiated	36	(45.6)
	Undifferentiated	43	(54.4)
cT			
	T1	0	(0)
	T2	2	(2.5)
	T3	14	(17.7)
	T4a	58	(73.4)
	T4b	5	(6.3)
cN			
	N0	12	(15.2)
	N1	31	(39.2)
	N2	29	(36.7)
	N3a	7	(8.9)
cM			
	M0	0	(0)
	M1	79	(100)
	P1	39	(49.4)
	CY1	31	(39.2)
	Lymph node	24	(30.4)
	H1	10	(12.7)
	Lung	4	(5.1)
	Ovary	3	(3.8)
Classification for stage IV GC			
	Category 1	23	(29.1)
	Category 2	17	(21.5)
	Category 3	33	(41.8)
	Category 4	6	(7.6)

GC, gastric cancer; IQR, interquartile range; No, number

^a^ Except as noted

Table 2 shows the details of pre-conversion chemotherapy. As 1st line chemotherapy, docetaxel, cisplatin, and S-1 was administered to 35 patients (44.3%); S-1 and cisplatin to 29 patients (36.7%); and other regimens to 15 patients (19.0%). Other regimens included oxaliplatin and S-1; docetaxel, oxaliplatin, and S-1; capecitabine and cisplatin; S-1 monotherapy; intraperitoneal chemotherapy-containing regimens; and trastuzumab-containing regimens. The reasons for termination of 1st line chemotherapy were adverse effects in 11 patients and tumor progression in 1 patient. The median time from the initiation of chemotherapy to CS was 141 days (interquartile range [IQR] 86–234). The median time from the initiation of chemotherapy to CS in IR and UR patients was 93 (IQR 81–139) and 160 (IQR 109–256), which was significantly different (P = 0.013).

**Table 2:** Chemotherapy type before surgery

Variable		No. of patients (%)^a^	
No. of regimens			
	1	67	(84.8)
	2	10	(12.7)
	4	2	(2.5)
Total number of courses, median (IQR)		4	(2–6)
Interval between initiation of chemotherapy and surgery (days), median (IQR)		141	(86–234)
First line regimen			
	DCS	35	(44.3)
	SP	29	(36.7)
	Others	15	(19.0)

No, number; DCS, docetaxel and cisplatin plus S-1; IQR, interquartile range; SP, S-1 and cisplatin

^a^ Except as noted

### Surgical and pathological findings

Table 3 shows surgical and pathological findings. Seventy-four patients (93.7%) underwent D2 or higher lymph node dissection. D1 dissection was performed in only one patient because macroscopic residual tumor was apparent intraoperatively (R2). Of the 18 patients with M1 lymph node metastasis, 14 underwent metastasectomy of the lymph node. R0 resection was achieved in 63 patients (79.7%), R1 in 11 (13.9%), and R2 in 5 (6.3%). The R0 resection rate was 100%, 94.1%, 60.6%, and 66.7% for patients in categories 1, 2, 3, and 4, respectively.

**Table 3:** Surgical and pathological findings

Variable		No. of patients (%)^a^	
Type of operative procedure			
	Total gastrectomy	44	(55.7)
	Distal gastrectomy	34	(43.0)
	Pancreatoduodenectomy	1	(1.3)
Lymph node dissection^b^			
	D1	1	(1.3)
	D1+	4	(5.1)
	D2	56	(70.9)
	D2+	18	(22.8)
Residual tumor			
	R0	63	(79.7)
	R1	11	(13.9)
	CY1	10	
	PM1	1	
	R2	5	(6.3)
Complications^c^			
	≥Grade 3	14	(17.7)
	≥Grade 2	17	(21.5)
	Abdominal abscess	5	
	Small bowel obstruction	3	
	Pneumonia	2	
	Anastomotic leakage	2	
	Others	5	
ypT			
	T0	3	(3.8)
	T1	7	(8.9)
	T2	10	(12.7)
	T3	31	(39.2)
	T4a	23	(29.1)
	T4b	5	(6.3)
ypN			
	N0	26	(32.9)
	N1	15	(19.0)
	N2	20	(25.3)
	N3a	8	(10.1)
	N3b	10	(12.7)
Histological response			
	Grade 0	7	(8.9)
	Grade 1	49	(62.0)
	Grade 2	20	(25.3)
	Grade 3	3	(3.8)
Adjuvant chemotherapy			
	Yes	74	(93.6)
	No	5	(6.4)

^a^ Except as noted.

^b^ Lymph node dissection assessed according to the Japanese gastric cancer treatment guidelines 2014 (ver. 4)

^c^ Complications assessed according to the Clavien–Dindo classification.

Pathological Grade 3 response was observed in 3 patients (4.1%) who had CY1 alone before preoperative chemotherapy. Of the 79 patients, 74 (93.7%) received postoperative chemotherapy. S-1-containing regimens were administered to 62 patients, and 44 of those received S-1 monotherapy. Eleven patients were treated with the same chemotherapeutic regimen both preoperatively and postoperatively.

### Overall survival and prognostic factors

**Fig. 1:**
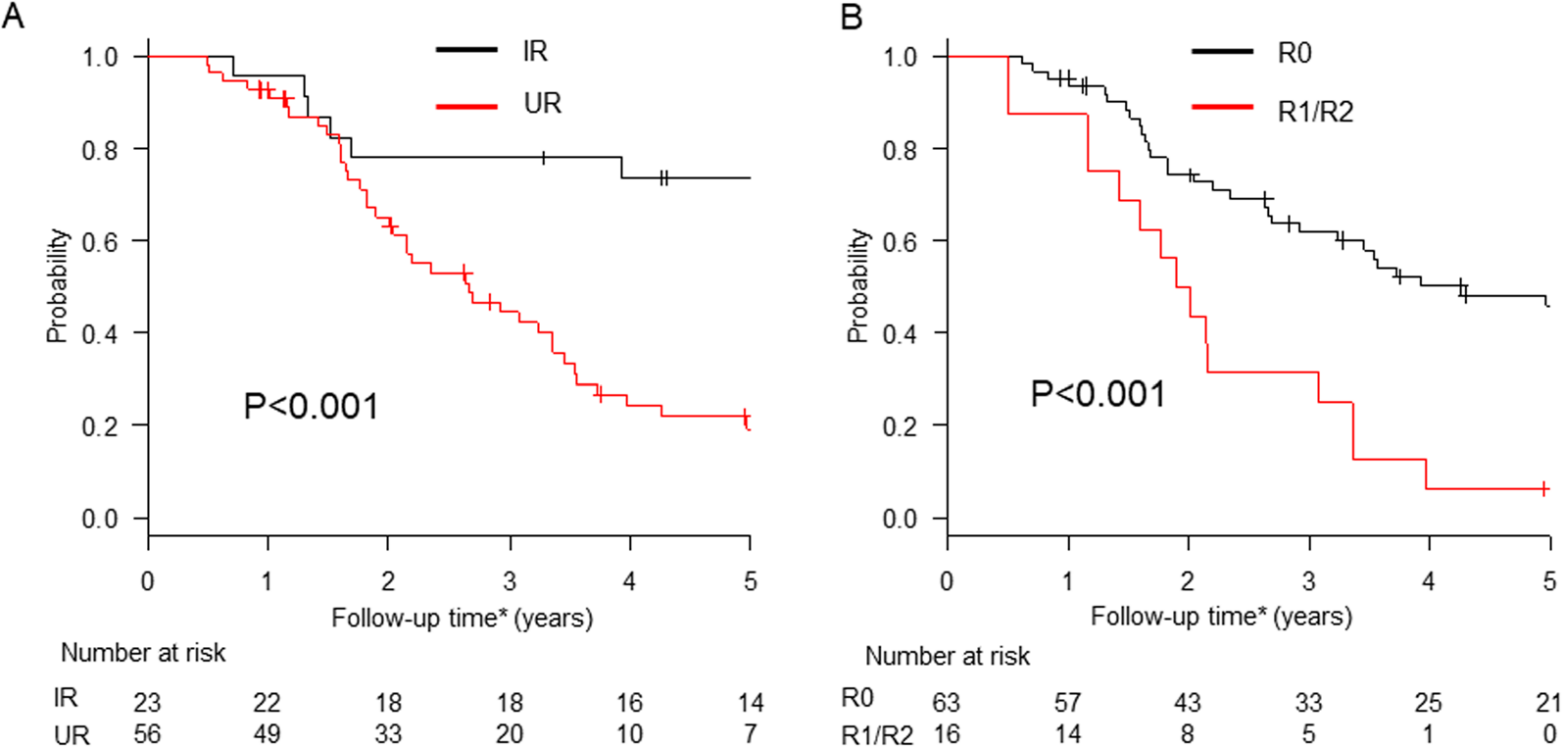
Kaplan–Meier survival curves for patients stratified by the initial status of stage IV disease (**a**) and the residual tumor status (**b**). IR = initially resectable disease, including patients with category 1 stage IV gastric cancer; UR = unresectable disease, including patients with category 2–4 stage IV gastric cancer. * Follow-up time: time from the start of initial chemotherapy to the date of death or of the last follow-up visit

Univariate survival analysis revealed that the initial resectability status (IR *vs* UR) of stage IV GC (*P* < 0.001) and residual tumor (*P* < 0.001) were significantly associated with OS (Table 4). The 3-year OS rates for patients with IR and UR were 78.3% and 44.5%, respectively (Fig. 1a), and the 3-year OS rates for patients with R0 and R1/2 resection were 61.8% and 31.3%, respectively (Fig. 1b). In the UR group, the 3- and 5-year OS rates of the patients with R1 resection due to CY1 were 20% and 0%, respectively. In multivariate Cox regression analysis, IR (HR 0.378, 95% CI 0.173–0.824, *P* = 0.014) and R0 resection (HR 0.439, 95% CI 0.227–0.847, *P* = 0.014) were significant independent predictors of favorable OS (Table 4).

**Table 4:** Univariate and multivariate Cox proportional hazards analyses of clinicopathological factors associated with overall survival

Factor	No. of patients	Univariate Analysis		Multivariate Analysis			
		3-year OS rate, %	P value	HR	95% CI	P value	
Sex			0.387				
Men	51	45.5					
Women	28	75.5					
Age			0.396				
< 60 years	26	64.7					
≥ 60 years	53	50.0					
Histological type			0.871				
Differentiated	36	59.5					
Undifferentiated	43	52.1					
cT			0.111				
Non-cT4	16	68.8					
cT4	63	51.5					
cN			0.775				
cN0	12	64.8					
cN1–N3	67	53.6					
Initial status of stage IV GC			< 0.001				
IR (Category 1)	23	78.3		0.378	0.173–0.824	0.014	
UR (Category 2–4)	56	44.5		1			
No. of regimens			0.558				
1	67	52.1					
≥ 2	12	72.7					
No. of cycles of chemotherapy			0.098				
< 5	45	56.7		0.977	0.524–1.821	0.942	
≥ 5	34	53.4		1			
Type of operative procedure			0.185				
Total gastrectomy	44	50.1					
Other	35	62.2					
Complication (Grade ≥ 3)			0.682				
Yes	14	41.7					
No	65	58.3					
Residual tumor			< 0.001				
R0	63	61.8		0.439	0.227–0.847	0.014	
R1/R2	16	31.3		1			
Histological response			0.406				
Grade 0/Grade 1	52	48.4					
Grade 2/Grade 3	22	70.3					

CI, confidence interval; GC, gastric cancer; HR, hazard ratio; IR, initially resectable disease; No., number; OS, overall survival; UR, unresectable disease

**Fig. 2:**
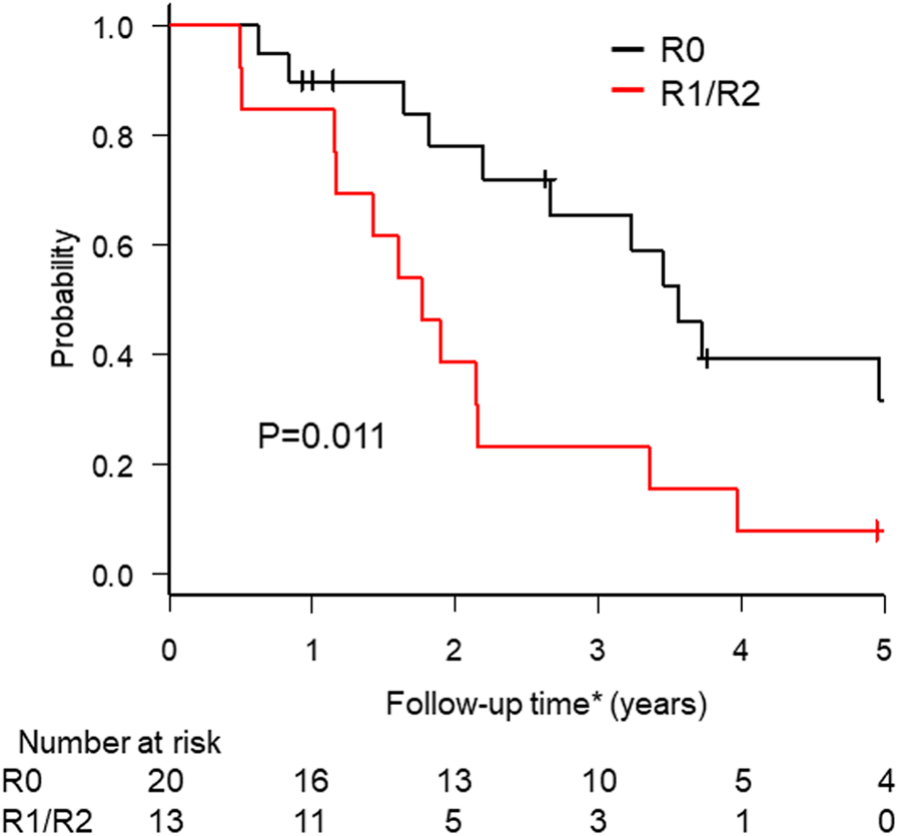
Kaplan–Meier survival curves for patients with category 3 stage IV gastric cancer stratified by the residual tumor status after conversion surgery (N = 33). Category 3 = macroscopic peritoneal dissemination without other distant metastasis. * Follow-up time: time from the start of initial chemotherapy to the date of death or of the last follow-up visit

We further evaluated OS after CS in subgroups according to the stage IV GC category. Figure 2 shows the Kaplan–Meier survival analysis for category 3 patients who had macroscopic peritoneal dissemination alone stratified by residual tumor status. OS after CS was significantly better for patients with R0 resection than for patients with R1/2 resection, with 3-year OS rates of 65.5% and 23.1%, respectively (*P* = 0.011). Among the category 2 patients who had technically and oncologically unresectable distant metastasis other than peritoneal dissemination, the 3-year OS rates were 48.5% and 0% and the MSTs were 31 months and 24 months for patients with R0 and R1 resection, respectively (*P* = 0.44, Additional file 1: Fig. S1a). None of the category 4 patients who had macroscopic peritoneal dissemination with other distant metastasis survived past the 3-year follow-up even after R0 resection (Additional file 1: Fig. S1b).

## Discussion

Although stage IV GC includes a mixture of tumor types with various metastatic characteristics and biology, recent advances in the use of chemotherapy have made CS a feasible and promising option for this disease. However, there is a paucity of data regarding the long-term outcomes and prognostic factors that enable the selection of stage IV GC patients who might be good candidates for CS. Our multicenter retrospective study demonstrated that IR stage IV GC (category 1) and R0 resection were significant predictors of favorable OS after CS. Among patients with initially UR disease, those with peritoneal dissemination alone (category 3), which is commonly uncurable and usually not considered for CS, had a favorable prognosis only after CS with R0 resection. Thus, the results of the present study may prove helpful in selecting patients with this highly heterogeneous disease who might respond to CS.

We found that the 3-year OS rate after CS was significantly better for the IR group (78.3%) than the UR group. Our definition of IR corresponded to category 1 defined by Yoshida et al., and included PAN No. 16a2 and/or 16b1 metastasis, a solitary liver metastasis of < 5 cm, and CY1 [13]. Kinoshita et al. reported that patients with potentially resectable disease, such as PAN No. 16a2 and/or 16b1 metastasis or fewer than three peripheral liver metastases, had significantly better prognosis than patients with initially UR disease [10]. However, CY1 differs from IR PAN or liver metastasis because it indicates the presence of microscopically unresectable disease. Although S-1 adjuvant chemotherapy following R1 resection due to CY1 has a relatively good outcome and prognosis, some studies reported favorable OS for patients with CY1 who underwent R0 resection after chemotherapy-induced conversion to a cytologically negative status (CY0) [11, 18–22]. In our study, all patients in the IR group with CY1 achieved CY0 and underwent R0 resection after chemotherapy, which contributed to the favorable prognosis in this group. The median time from the initiation of chemotherapy to CS in CY1 patients was 103 days, which was significantly shorter than duration of UR patients (data not shown). Yoshida et al. showed that the median duration of preoperative chemotherapy was 84 days for P0CY1 and R0 resection was achieved in 73.8% of patients with P0CY1 disease [11]. In CY1 GC, R0 resection rate may be higher even after a shorter duration of chemotherapy compared to UR. IR include stage IV GCs which are resectable before chemotherapy and which are potentially resectable after a shorter duration of chemotherapy compared to UR. Thus, we suggest that patients with IR are possible to be treated differently from those in UR.

We achieved a R0 resection rate of 79.7%, which is comparable to the range of 49.5–100% following CS for stage IV GC obtained in earlier studies [4, 5, 7, 10, 23]. The previous reports also showed that patients who achieved R0 resection had significantly better OS rates than patients who did not and found that R0 resection was an independent favorable prognostic factor for OS [4, 9]. Whether R1 resection is an acceptable outcome for CS is another issue. R1 resection is often observed in stage IV GC patients with CY1; indeed, this was true for 10 of the 11 patients who achieved R1 resection in the present study. The 5-year OS rate for patients with CY1 has been shown to be > 20% [18]. In our study, all of the patients with R1 resection due to CY1 were in the UR group and the 3- and 5-year OS rates were 20% and 0%, respectively, despite having received additional chemotherapy after CS (data not shown). The reason for this poor prognosis compared with the results of the previous report may be the presence of chemoresistant or poorly susceptible residual cancer cells after chemotherapy. These cancer cells are difficult to eliminate even with postoperative chemotherapy and may result in peritoneal dissemination. Surgeons should therefore confirm that cytology is negative before CS, especially in patients with UR disease. Another common reason for R1 resection is positive microscopic resection, and Solaini et al. reported that this was the only risk factor for recurrence after CS [4]. Therefore, R0 resection may be of great importance to observe improved survival in patients undergoing CS for stage IV GC.

Peritoneal dissemination is the most common noncurative factor in GC and is included in categories 3 and 4 stage IV GC. Patients with tumors in these categories are expected to have a poor prognosis even when R0 resection is accomplished [24]. However, some studies have shown favorable survival outcomes in category 3 patients who underwent CS [4, 5, 7]. For example, Leonardo et al. reported a 3-year OS rate of > 35% [4], Yamaguchi et al. reported an MST of 22.0 months for category 3 patients with CS and an MST of 33.3 months for those achieving R0 [5], and Chen et al. reported an MST for category 3 patients of 43.6 months [7]. In the present study, we observed an MST of 43.0 months and 3- and 5-year OS rates of 65.5% and 32.3%, respectively, for category 3 patients who underwent R0 resection, which is consistent with the results of previous reports. Fukuchi et al. reported that the combination of several noncurative factors, such as T4b, P1, H1, M1, and CY1, but not each factor individually, was associated with poor survival of patients who underwent CS [9]. These findings suggest that, although category 3 patients have a low R0 resection rate compared with category 1 or 2 patients, they are still good candidates for CS if R0 resection is possible.

In category 4, the conversion rate and the incidence of R0 resection was low, and the OS in the CS patients was significantly shorter than compared with the other categories [5, 7]. In the present study, the incidence of R0 resection was the same for category 4 and category 3 patients, but the 3-year OS rate was 0% for category 4 patients, even if R0 resection was achieved. CS is considered to be less effective for category 4 patients than for the other categories. However, Yoshida et al. demonstrated that in category 4 the 5-year OS was more than 50% and the prognosis was significantly longer in patients who underwent R0 resection than in those who underwent R1 or R2 resection, so R0 resection for category 4 is controversial [11].

The optimal timing of CS and the ideal number of chemotherapy courses before surgery in stage IV GC patients is controversial. In general, the best timing for CS is considered to be attainment of a partial or complete response to chemotherapy after a regimen of 4–6 courses on a 3–5-week cycle. This is based on the results of several studies showing that the median progression-free survival after each regimen for stage IV GC is about 6 months [25–27]. In the present study, the median number of chemotherapy cycles was 4 and the median time from initiation of chemotherapy to CS was 4.6 months, both of which were comparable to the results of previous studies [9, 28].

In this study, most of the patients received triplet regimens consisted of FU, platinum, and taxane or doublet regimens consisted of FU, and platinum. The phase III trail JCOG1013 did not demonstrate the superiority of docetaxel, cisplatin and S-1 compared with cisplatin and S-1 in chemotherapy-naive patients with advanced gastric cancer [29]. Chen GM et al. reported that the OS of the patients who underwent CS was not different between the patients who received triplet regimen and the patient who received doublet regimen [7]. Therefore, the difference between triplet regimen and doublet regimen is not expected to have a significant impact on OS. However, it is possible that a variety of regimens lead to differences in conversion rates. Yamaguchi T et al. reported that the incidence of conversion to CY0P0 in the patients with CY1 or P1a were 51.4% in the patients with docetaxel, cisplatin, and S-1 regimen and 37.4% in CS regimen [20]. Although the high chemotherapy response contributes to a survival benefit [30], high histological response did not improve survival in this study. In other CS studies, chemotherapy response before CS and histological response are also not one of prognostic factors [7–10]. This result supports the negative association between histological response and survival in the present study. Further research should be needed to reveal conversion rates by regimens and a potential association between chemotherapy response and survival.

This study has some limitations. First, this is a retrospective study with small sample size. Lage-scale multicenter randomized trails are needed to explore the role of CS and to determine the best treatment strategy for CS. Second, it was a multicenter retrospective study, and the indication and timing of CS differed between institutions. However, the treatment strategy for patients with stage IV GC who were given chemotherapy was to convert to surgery if R0 resection was possible, which was common to all participant institutions. Thus, there was little bias in patient indication of CS for stage IV GC. Third, the chemotherapy regimens were not unified, so the real impact of the regimen on CS outcomes was not clear. Forth, the study population included only patients who underwent CS, and whether CS had a survival benefit compared with chemotherapy alone is thus unclear. The MST after 1st line chemotherapy trails and CS is approximately 15 months [25–27]. The MST in the present study was much longer than 15 months, even for patients with UR or R1/R2. Moreover, most patients who underwent CS had histologically viable cancer cells in their resected specimens, and CS may have contributed to their improved survival by removing these cells. Nevertheless, we believe that our findings of this study provide useful information for the consideration of treatment strategies for stage IV GC.

## Conclusions

This study demonstrated that CS is a feasible treatment option for selected patients with stage IV GC. Patients with IR disease are good candidates for CS and are possible to be treated differently from those with UR disease. In CS for stage IV GC, achieving R0 resection could expect promising survival even for patients with UR disease. However, among patients with peritoneal dissemination, CS with R0 resection may contribute to favorable prognosis only in patients without other distant metastasis.

## Supplementary Information

**Additional file 1. Supplementary figure 1**. Kaplan–Meier survival curves for patients with category 2 (a, N = 17) and category 4 (b, N = 6) stage IV gastric cancer stratified by the residual tumor status after conversion surgery. * Follow-up time: time from the start of initial chemotherapy to the date of death or of the last follow-up visit Category 2 = distant metastasis that are regarded as technically and oncologically unresectable tumor without peritoneal dissemination. Category 4 = macroscopic peritoneal dissemination with other distant metastasis.

## Data Availability

All data generated or analyzed during this study are included in this article. Further inquiries can be directed to the corresponding author.

## Abbreviations

GC: Gastric cancer
CS: Conversion surgery
CT: Computed tomography
CY1: Positive for cancer on peritoneal cytology
PAN: Para aortic lymph node
IR: Initially resectable
UR: Unresectable
OS: Overall survival
CIs: Confidence intervals
IQR: Interquartile range
MST: Median survival time
DCS: Docetaxel and cisplatin plus S-1
SP: S-1 and cisplatin
HR: Hazard ratio
IR: Initially resectable disease
No.: Number
