# Supplementary material for: Conversion surgery for stage IV gastric cancer: a multicenter retrospective study
Source: BMC Surg. 2022 Dec 14;22:428. doi: 10.1186/s12893-022-01874-8 (PMC9749226; doi:10.1186/s12893-022-01874-8)
Supplement: Supplementary file 1 — Additional file 1. Supplementary figure 1. Kaplan–Meier survival curves for patients with category 2 (a, N = 17) and category 4 (b, N = 6) stage IV gastric cancer stratified by the residual tumor status after conversion surgery. * Follow-up time: time from the start of initial chemotherapy to the date of death or of the last follow-up visit Category 2 = distant metastasis that are regarded as technically and oncologically unresectable tumor without peritoneal dissemination. Category 4 = macroscopic peritoneal dissemination with other distant metastasis. [file 12893_2022_1874_MOESM1_ESM.pdf]

Supplementary Fig.

S1a

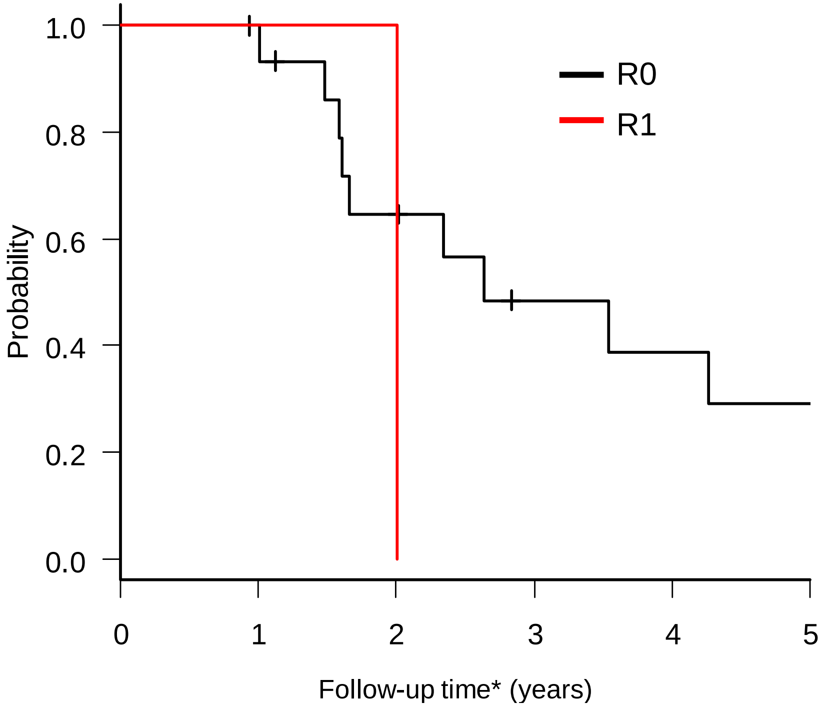


Number at risk

| | | | | | | |
|---|---|---|---|---|---|---|
| R0 | 16 | 15 | 9 | 5 | 4 | 3 |
| R1 | 1 | 1 | 1 | 0 | 0 | 0 |

S1b

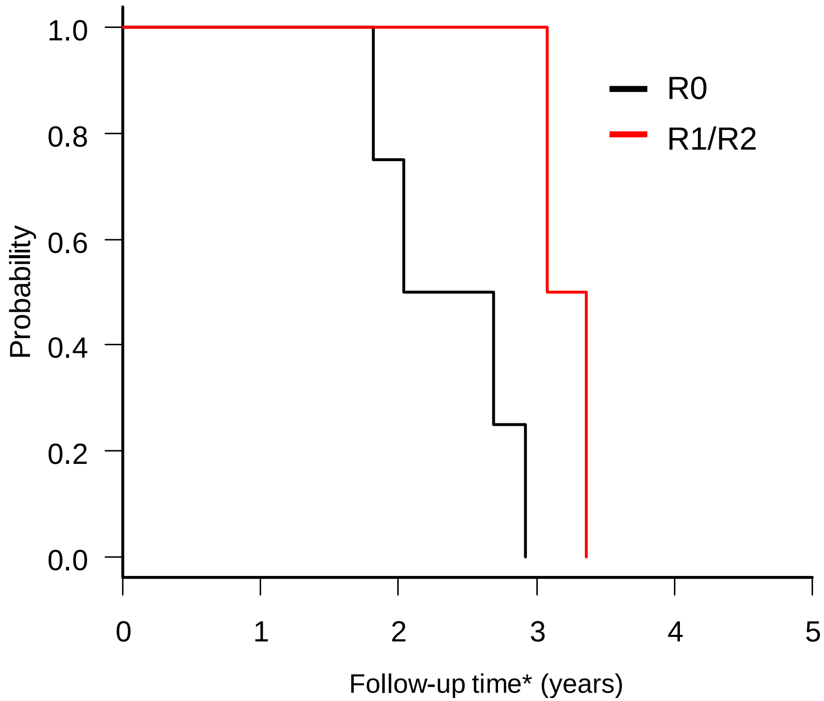


Number at risk

| | | | | | | |
|---|---|---|---|---|---|---|
| R0 | 4 | 4 | 3 | 0 | 0 | 0 |
| R1/R2 | 2 | 2 | 2 | 2 | 0 | 0 |

**Supplementary figure 1**. Kaplan–Meier survival curves for patients with category 2 (a, N = 17) and category 4 (b, N = 6) stage IV gastric cancer stratified by the residual tumor status after conversion surgery. * Follow-up time: time from the start of initial chemotherapy to the date of death or of the last follow-up visit

Category 2 = distant metastasis that are regarded as technically and oncologically unresectable tumor without peritoneal dissemination. Category 4 = macroscopic peritoneal dissemination with other distant metastasis.
